# Predictive Value of the Triglyceride to High-Density Lipoprotein Cholesterol Ratio for All-Cause Mortality and Cardiovascular Death in Diabetic Patients With Coronary Artery Disease Treated With Statins

**DOI:** 10.3389/fcvm.2021.718604

**Published:** 2021-07-21

**Authors:** Le Wang, Hongliang Cong, Jingxia Zhang, Yuecheng Hu, Ao Wei, Yingyi Zhang, Hua Yang, Libin Ren, Wei Qi, Wenyu Li

**Affiliations:** Department of Cardiology, Tianjin Chest Hospital, Tianjin, China

**Keywords:** triglyceride to high-density lipoprotein cholesterol ratio, type 2 diabetes, coronary artery disease, statin, all-cause mortality, cardiovascular death

## Abstract

**Background and Aims:** Studies have highlighted the role of the triglyceride to high-density lipoprotein cholesterol (TG/HDL-C) ratio on subsequent cardiovascular events. However, the association of the TG/HDL-C ratio with survival outcomes in diabetic patients with coronary artery disease (CAD) treated with statins remains unknown. This study aimed to assess the predictive value of the TG/HDL-C ratio for all-cause mortality and cardiovascular death in diabetic patients with CAD treated with statins.

**Methods:** The data of patients with type 2 diabetes and angiographically-confirmed CAD who were undergoing statin therapy and visited Tianjin Chest Hospital between January 2016 and September 2016 were retrospectively collected. The patients were categorized based on the baseline TG/HDL-C ratio tertile. Kaplan-Meier analysis and multivariate Cox proportional hazard regression were applied to assess the role of the TG/HDL-C ratio in predicting all-cause mortality and cardiovascular death.

**Results:** A total of 2,080 patients were included. During the 4-year follow-up, 209 patients died, 136 of whom from cardiovascular death. The Kaplan-Meier analyses showed that an increased TG/HDL-C ratio was associated with an increased risk of all-cause mortality (*P* < 0.001) and cardiovascular death (*P* < 0.001). The multivariate cox hazard regression analysis revealed a similar effect of the TG/HDL-C ratio on the risk of all-cause mortality (*P* = 0.046) and cardiovascular death (*P* = 0.009). The role of the TG/HDL-C ratio in predicting all-cause mortality and cardiovascular death was similar among all subgroups (*P* > 0.050). For all-cause mortality, the TG/HDL-C ratio significantly improved the C-statistic from 0.799 to 0.812 (*P* = 0.018), and the net reclassification index (NRI) and integrated discrimination index (IDI) were 0.252 (95% CI: 0.112–0.392; *P* < 0.001) and 0.012 (95% CI: 0.003–0.022; *P* = 0.012), respectively. Similarly, for cardiovascular death, the TG/HDL-C ratio significantly improved the C-statistic from 0.771 to 0.804 (*P* < 0.001), and the NRI and IDI were 0.508 (95% CI: 0.335–0.680; *P* < 0.001) and 0.033 (95% CI: 0.015–0.050; *P* < 0.001).

**Conclusion:** TG/HDL-C ratio might be useful for predicting all-cause mortality and cardiovascular death in diabetic patients with CAD treated with statins.

## Introduction

The role of diabetes mellitus (DM) on subsequent coronary artery disease (CAD) is well-illustrated (1), and studies have demonstrated that the use of statins could reduce the risk of major cardiovascular events (MACEs) in diabetic patients (2–5). However, patients with CAD have a higher prevalence of type 2 DM, and the risk of mortality remains high even in those treated with statins. The residual risk could be attributed to abnormal lipoprotein and lipid levels (6). Therefore, it is necessary that the lipid status be re-evaluated in diabetic patients with CAD treated with statins to identify those with higher residual risk such that tailored risk reduction strategies can be developed.

Dyslipidemia is characterized by elevated triglyceride (TG) and reduced dense high-density lipoprotein cholesterol particles levels, and lower high-density lipoprotein cholesterol (HDL-C) levels in diabetic patients (7, 8). Elevated TG and lower HDL-C are associated with poor prognosis in diabetic patients (9–12), but the use TG or HDL-C alone does not reflect the risk of atherosclerosis and cardiovascular disease (CVD) (13). The TG/HDL-C ratio may reflect the actual lipid profiles, and is considered an important marker of plasma atherosclerosis (14). Moreover, studies found that the TG/HDL-C ratio was an important predictor of insulin resistance and could evaluate the degree of abnormal glucose metabolism (15–17).

Numerous studies have reported a positive relationship between the TG/HDL-C ratio and hypertension (18–20), obesity (21), metabolic syndrome (22–24), hyperuricemia (25), and non-alcoholic fatty liver disease (26, 27). Moreover, an elevated TG/HDL-C ratio plays an important role on heart rate recovery after exercise (28), increased arterial stiffness (29, 30) and increased carotid atherosclerosis (31). Studies have indicated that the TG/HDL-C ratio should be considered as an important primary prevention cardiovascular risk factor, while the strength of the predictive value differs for patients undergoing various status (32–43). Furthermore, the predictive value of the TG/HDL-C ratio for all-cause mortality and cardiovascular death in diabetic patients with CAD treated with statins is unknown. This retrospective cohort study was therefore performed to assess the potential role of the TG/HDL-C ratio in the prediction of all-cause mortality and cardiovascular death in diabetic patients with CAD who were treated with statins.

## Methods

### Study Population

Patients who were admitted to Tianjin Chest Hospital between January 2016 and September 2016 were recruited in this retrospective cohort study. A total of 2,678 patients with T2DM and angiographically-confirmed CAD were included. CAD comprised stable angina pectoris (SAP) and acute coronary syndrome (ACS). ACS included unstable angina pectoris, non-ST-segment elevation myocardial infarction, and ST-segment elevation myocardial infarction (STEMI). Patients were excluded if they met any of the following criteria: (1) aged < 18.0 or >80.0 years (*n* = 72), (2) severe valvular heart disease or congenital heart disease (*n* = 34), (3) alanine aminotransferase level > 3-fold greater than the normal upper limit (*n* = 15), (4) serum creatinine level > 1.5-fold greater than the normal upper limit (*n* = 96), (5) hyperthyroidism or hypothyroidism (*n* = 16), (6) incomplete clinical data (*n* = 75), and (7) not treated with statins (*n* = 99). The remaining 2,271 patients were recruited, and 2,080 patients with full clinical data after 4-year follow-up were included in the final analysis. The patients were categorized based on the tertiles of the baseline TG/HDL-C ratio, as follows: tertile 1 (*n* = 693, TG/HDL-C ratio ≤ 1.20), tertile 2 (*n* = 693, 1.20 < TG/HDL-C ratio ≤ 1.92), and tertile 3 (*n* = 694, TG/HDL-C ratio > 1.92). The study was approved by the Ethical Committee of Tianjin Chest Hospital (NO:2021LW-006), and the need to obtain informed consent requirement was waived as the study comprised a retrospective analysis of clinical data.

### Data Collection and Definitions

Baseline demographic characteristics, clinical presentation, cardiac function, extent of lesion, treatment strategy, laboratory findings at fasting status, and medication data at discharge were collected from medical records and the data managers were blinded to the study purpose. The demographic characteristics included age; sex ratio; duration of diabetes; smoker proportion; hypertension; prior myocardial infarction (MI), percutaneous coronary intervention (PCI), coronary artery bypass graft (CABG), or stroke; and body mass index (BMI). The cardiac function included left ventricle ejection fraction (LVEF). The clinical presentation included SAP and ACS, and the extent of lesion included left main disease and multi-vessel disease (>2 vessels with ≥50% diameter stenosis in major coronary arteries). The treatment strategies included medical therapy, PCI, and CABG. Laboratory findings included fasting plasma glucose (FPG), hemoglobin A1c (HbA1c), total cholesterol (TC), TG, low-density lipoprotein cholesterol (LDL-C), HDL-C, the TG/HDL-C ratio, serum uric acid, high-sensitivity C-reactive protein (hs-CRP), and estimated glomerular filtration rate (eGFR). The medications at discharge included aspirin, clopidogrel/ticagrelor, β-blocker, angiotensin II coenzyme inhibitor (ACEI) or angiotensin II receptor blocker (ARB), calcium channel blocker (CCB), nitrate, and insulin.

### Endpoints and Follow-Up Data

The investigated endpoints included all-cause mortality and cardiovascular death. All-cause mortality was defined as death from any cause, and cardiovascular death was defined as death caused by acute MI, heart failure, cardiac arrhythmia, or stroke. The follow-up information was collected by telephone or electronic medical record review.

### Statistical Analysis

Continuous variables are presented as the mean [standard deviation (SD)] and median (interquartile) based on data distribution, and the differences among groups were compared using an analysis of variance or the Kruskal-Wallis test. Categorical variables are presented as frequencies and proportions, and the differences among groups were compared using the Chi-square or Fisher's exact tests. The association between the TG/HDL-C ratio and subsequent all-cause mortality and cardiovascular death were assessed using Kaplan-Meier analysis and the log-rank test. Multivariate Cox regression analysis was performed to identify the independent predictors of all-cause mortality and cardiovascular death. All the variables in Table 1 were listed in univariate model and then were introduced into the multivariate model if the *P*-value was <0.10. The possible factors included age, duration of diabetes, hypertension, previous MI, previous PCI, previous stroke, LVEF, left main disease, multi-vessel disease, FPG, TC, LDL-C, uric acid, hs-CRP, and eGFR. Sensitivity analyses were performed for all-cause mortality and cardiovascular death by sequential adjustment of potential confounders. The C-statistics, net reclassification improvement (NRI), and integrated discrimination improvement (IDI) were applied to assess the incremental predictive value of the TG/HDL-C ratio over the established model (including age, duration of diabetes, previous PCI, LVEF, left main disease, multi-vessel disease, FPG, and eGFR). The optimal cut-off values of the TG/HDL-C ratio for predicting all-cause mortality and cardiovascular death were determined using receiver operating characteristic (ROC) curves. Subgroup analyses for all-cause mortality and cardiovascular death were conducted according to sex (male or female), smoker (yes or no), BMI (≤ 28 or >28 kg/m^2^), duration of DM (≤ 10 or >10 years), ACS (yes or no), HbA1c (≤ 7.0 or >7.0%), LDL-C (≤ 1.8 or >1.8 mmol/L), insulin treatment (yes or no), and revascularization (yes or no). The differences between subgroup analyses were also compared using the interaction *t*-test. All *P*-values are two-sided, and the inspection level was 0.050. The statistical analyses in this study were performed using SPSS version 20.0 (IBM Corp, Armonk, New York) and SAS version 9.1.3 (Cary, NC, USA).

**Table 1:** Baseline characteristics of included patients.

**Clinical characteristics**	**Tertile 1**	**Tertile 2**	**Tertile 3**	***P*-value**
	***N* = 693**	***N* = 693**	***N* = 694**	
Age, years	66.2 ± 6.7	66.2 ± 6.9	66.1 ± 6.7	0.870
Female	293 (42.3)	302 (43.6)	318 (45.8)	0.405
Duration of diabetes	9.5 ± 7.9	9.8 ± 7.5	9.9 ± 7.7	0.636
Smoker	265 (38.2)	289 (41.7)	267 (38.5)	0.337
Hypertension	529 (76.3)	531 (76.6)	525 (75.6)	0.909
Previous MI	80 (11.5)	86 (12.4)	91 (13.1)	0.674
Previous PCI	153 (22.1)	130 (18.8)	132 (19.0)	0.228
Previous CABG	24 (3.5)	25 (3.6)	32 (4.6)	0.479
Previous stroke	157 (22.7)	143 (20.6)	134 (19.3)	0.303
BMI, kg/m^2^	25.3 ± 2.9	25.5 ± 2.7	25.7 ± 2.8	0.020
LVEF	58 ± 8	58 ± 9	58 ± 9	0.193
Clinical presentation				0.353
SAP	131 (18.9)	111 (16.0)	118 (17.0)	
ACS	562 (81.1)	582 (84.0)	576 (83.0)	
Left main disease	69 (10.0)	78 (11.3)	71 (10.2)	0.707
Multi-vessel disease	561 (81.0)	563 (81.2)	570 (82.1)	0.841
Treatment strategy				0.880
MT	219 (31.6)	202 (29.1)	214 (30.8)	
PCI	399 (57.6)	410 (59.2)	406 (58.5)	
CABG	73 (10.5)	79 (11.4)	73 (10.5)	
**Laboratory findings**				
FPG, mmol/L	7.9 ± 2.9	8.0 ± 3.0	8.2 ± 3.3	0.077
HbA1c, %	7.4 ± 1.3	7.5 ± 1.4	7.7 ± 1.6	0.002
TC, mmol/L	4.58 ± 1.16	4.45 ± 1.07	4.31 ± 1.10	<0.001
TG, mmol/L	1.01 (0.81–1.21)	1.52 (1.28–1.79)	2.41 (1.94–3.06)	<0.001
LDL-C, mmol/L	2.96 ± 1.02	2.96 ± 0.94	2.85 ± 0.95	0.053
HDL-C, mmol/L	1.20 ± 0.29	1.01 ± 0.23	0.92 ± 0.22	<0.001
TG/HDL-C ratio	0.88 ± 0.21	1.54 ± 0.20	3.09 ± 1.62	<0.001
Uric acid, umol/L	305.4 ± 93.4	321.7 ± 92.0	331.1 ± 100.3	<0.001
hs-CRP, mg/L	1.50 (0.59–4.76)	1.83 (0.79–4.64)	2.08 (0.94–4.82)	<0.001
eGFR, mL/min	94.0 ± 24.8	92.3 ± 23.8	89.6 ± 24.6	0.003
**Medications at discharge**				
Aspirin	668 (96.4)	673 (97.1)	675 (97.3)	0.605
Clopidogrel/Ticagrelor	559 (80.7)	574 (82.8)	581 (83.7)	0.307
β-blocker	450 (64.9)	461 (66.5)	456 (65.7)	0.824
ACEI/ARB	404 (58.3)	381 (55.0)	407 (58.6)	0.313
CCB	174 (25.1)	199 (28.7)	203 (29.3)	0.172
Nitrate	382 (55.1)	379 (54.7)	391 (56.3)	0.814
Insulin	284 (41.0)	280 (40.4)	273 (39.3)	0.818

*Data are expressed as mean ± SD, medians with interquartile ranges or percentage. MI, myocardial infarction; PCI, percutaneous coronary intervention; CABG, coronary artery bypass graft; BMI; body mass index; LVEF, left ventricle ejection fraction; SAP, stable angina pectoris; ACS, acute coronary syndrome; MT, medical therapy; FPG, fasting plasma glucose; HbA1c, Hemoglobin A1c; TC, total cholesterol; TG, triglycerides; LDL-C, low-density lipoprotein cholesterol; HDL-C, high-density lipoprotein cholesterol; hs-CRP, high-sensitivity C-reactive protein; eGFR, estimated glomerular filtration rate; ACEI, angiotensin II coenzyme inhibitor; ARB, angiotensin II receptor blocker; CCB, calcium channel blocker; SD, standard deviation*.

## Results

### Baseline Characteristics

A total of 2,080 diabetic patients with CAD who were treated with statins were selected for analysis. The baseline characteristics of the patients in the three TG/HDL-C ratio categories are summarized in Table 1. Most variables did not significantly differ among the groups, including age; sex ratio; duration of diabetes; smoker proportion; hypertension; prior MI, PCI, CABG, or stroke; LVEF; clinical presentation; left main disease; multi-vessel disease; treatment strategy; FPG; LDL-C; aspirin; clopidogrel/ticagrelor; β-blocker; ACEI or ARB; CCB; nitrate; and insulin (*P* > 0.050). However, there were significant differences among the three groups in BMI (*P* = 0.020), HbA1c (*P* = 0.002), TC (*P* < 0.001), TG (*P* < 0.001), HDL-C (*P* < 0.001), TG/HDL-C ratio (*P* < 0.001), serum uric acid (*P* < 0.001), hs-CRP (*P* < 0.001), and eGFR (*P* = 0.003).

### TG/HDL-C Ratio and All-Cause Mortality

**Figure 1:**
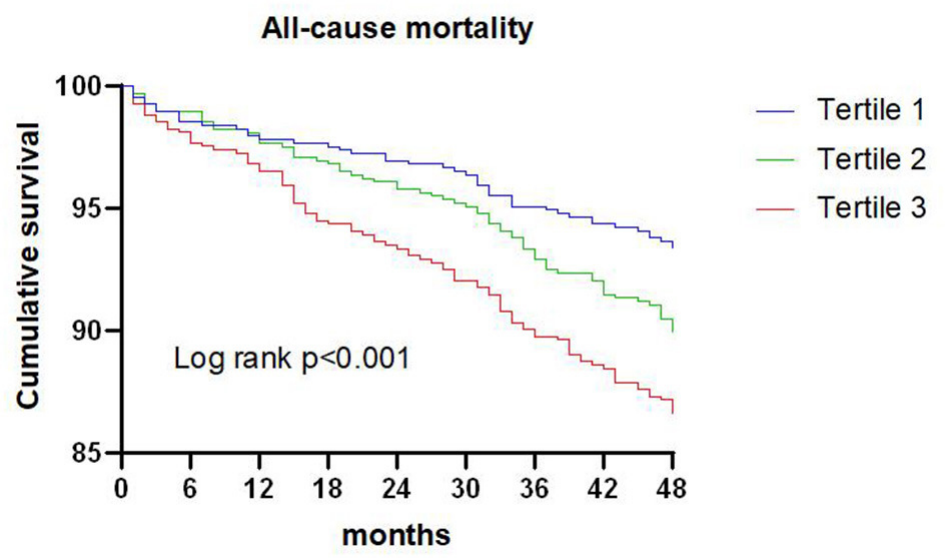
Kaplan-Meier survival curve for all-cause mortality across triglyceride to high density lipoprotein-C ratio tertiles.

A total of 209 patients died during the 4-year follow-up, and the proportions of all-cause mortality in tertiles 1, 2, and 3 were 6.6, 10.1, and 13.4%, respectively. Kaplan-Meier analysis indicated that an increased TG/HDL-C ratio was associated with an increased risk of all-cause mortality (*P* < 0.001; Figure 1). The Cox proportional hazard regression indicated that an increased TG/HDL-C ratio tertile was associated with an increased risk of all-cause mortality, irrespective of whether the unadjusted (*P* < 0.001) or adjusted (*P* = 0.046) was used. Moreover, per SD increment in the TG/HDL-C ratio was associated with an increased risk of all-cause mortality in both the unadjusted model (HR: 1.17; 95% CI: 1.10–1.24; *P* < 0.001) and the adjusted model (HR: 1.20; 95% CI: 1.11–1.30; *P* < 0.001) (Table 2). The role of the TG/HDL-C ratio in predicting the risk of all-cause mortality was robust after sequential adjustment for potential confounders (Table 3).

**Table 2:** Cox regression models in the prediction of all-cause mortality and cardiovascular death according to the triglyceride to high density lipoprotein-C ratio at baseline.

**Endpoint**	**Events, *n*/total (%)**	**Crude HR (95% CI)**	**Crude *P*-value**	**Adjusted HR (95% CI)**	**Adjusted *P-*value**
All-cause mortality			<0.001		0.046
Tertile 1	46/693 (6.6)	1.00 (reference)		1.00 (reference)	
Tertile 2	70/693 (101.)	1.54 (1.06–2.23)		1.19 (0.81–1.75)	
Tertile 3	93/694 (13.4)	2.09 (1.47–2.98)		1.52 (1.05–2.19)	
Per 1-SD		1.17 (1.10–1.24)	<0.001	1.20 (1.11–1.30)	<0.001
Cardiovascular death			<0.001		0.009
Tertile 1	27/693 (3.9)	1.00 (reference)		1.00 (reference)	
Tertile 2	43/693 (6.2)	1.65 (1.02–2.67)		1.40 (0.85–2.30)	
Tertile 3	66/694 (9.5)	2.55 (1.63–4.00)		2.01 (1.27–3.21)	
Per 1-SD		1.22 (1.16–1.29)	<0.001	1.27 (1.19–1.36)	<0.001

*Adjusted variables were age, duration of diabetes, hypertension, previous MI, previous PCI, previous stroke, LVEF, left main disease, multi-vessel disease, FPG, TC, LDL, Uric acid, hs-CRP, eGFR. HR, hazard ratio; CI, confidential interval; SD, standard deviation*.

**Table 3:** Sensitivity analysis of the association of the triglyceride to high density lipoprotein-C ratio per 1 standard deviation with mortality after separate adjustment for each of the other significant variables.

	**Multivariable analysis for all-cause mortality**			**Multivariable analysis for cardiovascular death**		
**Adjustment**	**HR for TG/HDL-C per**			**HR for TG/HDL-C per**		
**Variable**	**1-SD**	**95%CI**	***P*-value**	**1-SD**	**95%CI**	***P*-value**
Age	1.20	1.13–1.28	<0.001	1.26	1.19–1.33	<0.001
Smoker	1.17	1.10–1.24	<0.001	1.23	1.16–1.30	<0.001
Duration of diabetes	1.17	1.10–1.24	<0.001	1.23	1.16–1.30	<0.001
Hypertension	1.19	1.11–1.26	<0.001	1.25	1.18–1.33	<0.001
Previous MI	1.17	1.10–1.24	<0.001	1.22	1.16–1.29	<0.001
Previous PCI	1.18	1.11–1.25	<0.001	1.24	1.17–1.32	<0.001
Previous stroke	1.17	1.10–1.24	<0.001	1.22	1.16–1.29	<0.001
LVEF	1.20	1.12–1.27	<0.001	1.26	1.19–1.34	<0.001
Left main disease	1.17	1.10–1.24	<0.001	1.23	1.16–1.30	<0.001
Multi-vessel disease	1.17	1.10–1.24	<0.001	1.22	1.16–1.29	<0.001
FPG	1.17	1.10–1.24	<0.001	1.23	1.17–1.30	<0.001
TC	1.16	1.09–1.24	<0.001	1.22	1.16–1.29	<0.001
LDL-C	1.16	1.10–1.24	<0.001	1.23	1.16–1.30	<0.001
Uric acid	1.15	1.08–1.23	<0.001	1.21	1.15–1.29	<0.001
hs-CRP	1.17	1.10–1.25	<0.001	1.23	1.17–1.30	<0.001
eGFR	1.15	1.08–1.23	<0.001	1.21	1.14–1.28	<0.001

*MI, myocardial infarction; PCI, percutaneous coronary intervention; LVEF, left ventricle ejection fraction; FPG, fasting plasma glucose; HbA1c, Hemoglobin A1c; TC, total cholesterol; LDL-C, low-density lipoprotein cholesterol; hs-CRP, high-sensitivity C-reactive protein; eGFR, estimated glomerular filtration rate; TG, triglycerides; HDL-C, high-density lipoprotein cholesterol; HR, hazard ratio; CI, confidential interval; SD standard deviation*.

ROC analysis indicated that the optimal cutoff value of the TG/HDL-C ratio for predicting all-cause mortality was 1.77 (sensitivity: 53.1% and specificity: 62.8%), and the area under the curve (AUC) was 0.601 (95% CI: 0.561–0.640; *P* < 0.001). Adding the TG/HDL-C ratio to the model of established risk factors including age, duration of diabetes, previous PCI, LVEF, left main disease, multi-vessel disease, FBG, and eGFR improved the prediction of all-cause mortality in terms of the C-statistic (from 0.799 to 0.812; *P* = 0.018), and the NRI and IDI were 0.252 (95% CI: 0.112–0.392; *P* < 0.001) and 0.012 (95% CI: 0.003–0.022; *P* = 0.012), respectively (Table 4).

**Table 4:** Evaluation of predictive models for all-cause mortality and cardiovascular death.

**Endpoint**		**C-Statistic**	***P*-value**	**NRI (95%CI)**	***P*-value**	**IDI (95%CI)**	***P*-value**
All-cause mortality	Original model	0.799 (0.766–0.833)	Ref.		Ref.		Ref.
	Original model+ TG/HDL-C ratio	0.812 (0.780–0.844)	0.018	0.252 (0.112–0.392)	<0.001	0.012 (0.003–0.022)	0.012
Cardiovascular death	Original model	0.771 (0.728–0.814)	Ref.		Ref.		Ref.
	Original model+ TG/HDL-C ratio	0.804 (0.765–0.844)	<0.001	0.508 (0.335–0.680)	<0.001	0.033 (0.015–0.050)	<0.001

*Original model included age, duration of diabetes, previous PCI, LVEF, left main disease, multi-vessel disease, FPG and eGFR. TG, triglycerides; HDL-C, high-density lipoprotein cholesterol; NRI, net reclassification improvement; IDI, integrated discrimination improvement; CI, confidential interval*.

The results of subgroup analyses for all-cause mortality are illustrated in Table 5. An elevated TG/HDL-C ratio was associated with an increased risk of all-cause mortality in all subgroups, and the differences between subgroups were not significant based on sex (*P* = 0.985), smoker (*P* = 0.173), BMI (*P* = 0.741), duration of DM (*P* = 0.090), ACS (*P* = 0.438), HbA1c (*P* = 0.524), LDL-C (*P* = 0.788), insulin treatment (*P* = 0.265), and revascularization (*P* = 0.780).

**Table 5:** All-cause mortality and cardiovascular death in the various patient subgroups.

**Variable**	**Subgroups**	**All-cause mortality**				**Cardiovascular death**			
		**≤1.77**	**>1.77**	**HR (95%CI)**	***P* for interaction**	**≤1.57**	**>1.57**	**HR (95%CI)**	***P* for interaction**
All patient	Total	99/1,274	110/806	1.821 (1.388–2.389)		36/1,083	100/997	3.124 (2.135–4.573)	
Sex	Women	45/555	47/358	1.661 (1.104–2.500)	0.985	15/462	49/451	3.453 (1.936–6.156)	0.552
	Men	54/719	63/448	1.956 (1.360–2.813)		21/621	51/546	2.867 (1.725–4.766)	
Smoker	No	61/770	57/489	1.498 (1.044–2.150)	0.173	21/646	59/613	3.041 (1.848–5.003)	0.537
	Yes	38/504	53/317	2.360 (1.556–3.580)		15/437	41/384	3.262 (1.805–5.893)	
BMI (kg/m^2^)	≤ 28	79/1,058	91/655	1.930 (1.428–2.609)	0.741	33/905	83/808	2.918 (1.949–4.367)	0.285
	>28	20/216	19/151	1.409 (0.752–2.640)		3/178	17/189	5.504 (2.613–8.783)	
Duration of DM (years)	≤ 10	53/773	63/491	1.933 (1.341–2.785)	0.090	17/654	62/610	4.052 (2.369–6.929)	0.442
	>10	46/501	47/315	1.697 (1.130–2.548)		19/429	38/387	2.291 (1.321–3.973)	
ACS	No	16/241	15/119	1.973 (0.975–3.990)	0.438	4/241	12/119	6.312 (2.036–9.587)	0.346
	Yes	83/1,033	95/687	1.783 (1.328–2.394)		32/842	88/878	2.726 (1.819–4.085)	
HbA1c (%)	≤ 7.0	41/584	46/336	2.016 (1.323–3.071)	0.524	14/499	43/421	3.803 (2.081–6.952)	0.697
	>7.0	58/690	64/470	1.682 (1.179–2.400)		22/584	57/576	2.700 (1.651–4.415)	
LDL-C (mmol/L)	≤ 1.8	13/149	16/118	1.608 (0.773–3.343)	0.788	8/136	11/131	3.853 (1.075–13.810)	0.345
	>1.8	86/1,125	94/688	1.854 (1.384–2.483)		51/947	89/866	3.064 (2.055–4.568)	
Insulin treatment	No	51/751	66/492	2.065 (1.433–2.976)	0.265	18/632	61/611	3.616 (2.136–6.112)	0.502
	Yes	48/523	44/314	1.566 (1.040–2.357)		18/451	39/386	2.641 (1.511–4.617)	
Revascularization	No	31/393	35/247	1.876 (1.157–3.042)	0.780	9/327	37/313	4.432 (2.139–9.183)	0.476
	Yes	68/881	75/559	1.796 (1.293–2.494)		27/726	63/684	2.673 (1.703–4.195)	

*BMI, body mass index; DM, diabetes mellitus; ACS, acute coronary syndrome; HbA1c, Hemoglobin A1c; LDL-C, low-density lipoprotein cholesterol; HR, hazard ratio; CI, confidential interval*.

### TG/HDL-C Ratio and Cardiovascular Death

**Figure 2:**
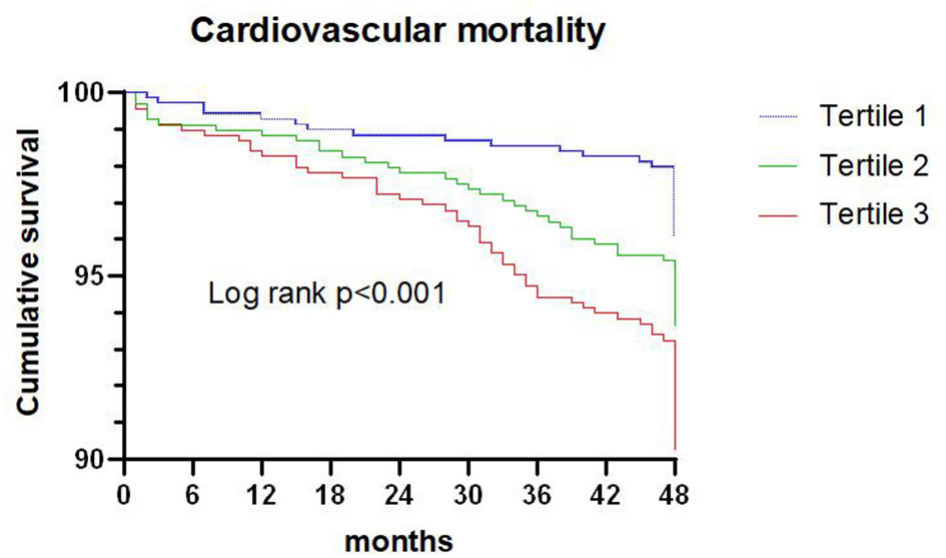
Kaplan-Meier survival curve for cardiovascular death across triglyceride to high density lipoprotein-C ratio tertiles.

A total of 136 patients died from cardiovascular death during the 4-year follow-up, and the proportion of cardiovascular death in tertiles 1, 2, and 3 were 3.9, 6.2, and 9.5%, respectively. Kaplan-Meier analysis suggested that the risk of cardiovascular death was significantly increased with an elevated TG/HDL-C ratio (*P* < 0.001; Figure 2).

Cox proportional hazard regression indicated that an increased TG/HDL-C ratio tertile was associated with an increased risk of cardiovascular death in both the unadjusted model (*P* < 0.001) and the adjusted model (*P* = 0.009). Furthermore, the risk of cardiovascular death was significantly increased per SD increment in the TG/HDL-C ratio in both the unadjusted model (HR: 1.22; 95% CI: 1.16–1.29; *P* < 0.001) and the adjusted model (HR: 1.27; 95% CI: 1.19–1.36; *P* < 0.001) (Table 2). Sensitivity analysis revealed that the association between the TG/HDL-C ratio and the risk of cardiovascular death was robust and not altered by sequential adjustment for potential confounders (Table 3).

ROC analysis indicated that the optimal cutoff value of the TG/HDL-C ratio for predicting cardiovascular death was 1.57 (sensitivity: 74.3% and specificity: 53.8%), with an AUC of 0.672 (95% CI: 0.625–0.718; *P* < 0.001). Adding the TG/HDL-C ratio to the established model improved the prediction of cardiovascular death in terms of the C-statistic (from 0.771 to 0.804; *P* < 0.001), and the NRI and IDI were 0.508 (95% CI: 0.335–0.680; *P* < 0.001) and 0.033 (95% CI: 0.015–0.050; *P* < 0.001), respectively (Table 4).

The results of the subgroup analyses for cardiovascular death based on pre-defined variables are shown in Table 5. An elevated TG/HDL-C ratio was associated with an increased risk of cardiovascular death in all subgroups, and sex (*P* = 0.552), smoker (*P* = 0.537), BMI (*P* = 0.285), duration of DM (*P* = 0.442), ACS (*P* = 0.346), HbA1c (*P* = 0.697), LDL-C (*P* = 0.345), insulin treatment (*P* = 0.502), and revascularization (*P* = 0.476) did not affect the role of TG/HDL-C ratio in predicting the risk of cardiovascular death.

## Discussion

This study systematically analyzed the predictive value of the TG/HDL-C ratio for subsequent all-cause mortality and cardiovascular death in diabetic patients with CAD who were treated with statins. An elevated TG/HDL-C ratio was associated with an increased risk of all-cause mortality and cardiovascular death. Sensitivity analyses indicated that the role of TG/HDL-C ratio in predicting subsequent all-cause mortality and cardiovascular death was robust and not altered by sequential adjusted potential confounders. Furthermore, adding the TG/HDL-C ratio to the established model resulted in a significant enhancement of the predictive value. The risk of all-cause mortality and cardiovascular death was significantly increased when the TG/HDL-C ratio was increased in all subgroups, and these associations were not affected by sex, smoker, BMI, duration of DM, ACS, HbA1c, LDL-C, insulin treatment, or revascularization. The above results indicate that the TG/HDL-C ratio is a marker of poor prognosis even in the era of statin treatment and may contribute to the early identification of high-risk diabetic patients and CAD. Furthermore, routine TG/HDL-C ratio calculation may further improve risk stratification for all-cause mortality and cardiovascular death.

LDL-C plays a key role in the development and progression of atherosclerotic CVD (ASCVD) and statins are the first-line therapy for lowering LDL-C levels to reduce ASCVD risk. However, diabetic patients with CAD remain at high cardiovascular risk even after LDL-C reduction, which indicates that there are residual cardiovascular risk factors other than LDL-C. One study found that diabetic patients treated with statins had a high prevalence of persistent atherogenic dyslipidemia (13). Elevated TG levels and lower HDL-C levels, as typical lipid features of diabetes, are considered to indicate atherogenic dyslipidemia in diabetic patients (44, 45). However, the levels of TG and HDL-C are mutually independent, and the single lipid parameter could not reflect the actual status of plasma atherogenicity and CVD risk in the absence of insulin resistance (13). Therefore, the TG/HDL-C ratio could reflect TG and HDL-C simultaneously, and is regarded as a better marker in primary and secondary prevention of CVD (34, 36, 46). A study conducted by Edwards et al. suggested that the TG/HDL-C ratio has better predictive value for mortality than that of individual lipid parameters (47). Furthermore, a high TG/HDL-C ratio may strongly predict the extent of coronary lesions (48, 49). Moreover, the TG/HDL-C ratio is significantly related to vulnerable plaque features in diabetic patients treated with statins (50). Routine lipid examinations do not reflect the actual compositional changes of lipid parameters in diabetic patients with CAD. Therefore, evaluation of the TG/HDL-C ratio may have great clinical significance with regards to risk stratification for diabetic patients with CAD who are treated with statins.

Although previous studies have demonstrated the role of the TG/HDL-C ratio in predicting adverse cardiovascular events in patients with CAD (51–55), the potential role of TG/HDL-C ratio as a prognostic marker for patients with diabetes is still debated. The Swedish National Diabetes Register found that elevated TG/HDL-C ratio could increase the risk of CVD independent of the LDL-C level in obese T2DM patients (56). Yang et al. reported that the TG/HDL-C ratio was an important predictor of MACEs in patients with diabetes and CAD (42). Contrary to these studies, several other studies did not find significant associations between the TG/HDL-C ratio and the prognosis of T2DM. Tohidi et al. demonstrated that the TG/HDL-C ratio was not an independent predictor of cardiovascular events in diabetic patients without CVD (57). The sub analysis of the Management of Elevated Cholesterol in the Primary Prevention Group of Adult Japanese (MEGA) study was not able to establish an independent association between TG/HDL ration and CVD risk in patients with DM and without history of CVD (43). The potential reasons for this discrepancy could be the variation in definition of endpoints, patient characteristics among studies.

This study is the first to focus on the role of the TG/HDL-C ratio in the prediction of prognosis in diabetic patients with CAD who were treated with statins. Compared with previous studies focusing on patients with diabetes or CAD, this large cohort study included higher risk patients with a higher prevalence of a history of CVD. This study demonstrated that an elevated TG/HDL-C ratio was associated with poor prognosis in diabetic patients with CAD treated with statins. Although higher TG/LDL-C ratio were relevant for chronic kidney disease (CKD) in patients with diabetes (58), TG/LDL-C ratio remained a significant and independent predictor of all-cause mortality and cardiovascular death after adjustment for potential confounders including renal function measures (eGFR). This finding suggested that the association between TG/HDL-C ratio and the risk of mortality might not be mediated by the presence of kidney dysfunction. These associations were persistent in sensitivity and subgroup analyses. An elevated TG/HDL-C ratio was still associated with an increased risk of mortality in patients with LDL-C levels of ≤ 1.80 mmol/L, suggesting that the ratio may explain part of the residual cardiovascular risk. The use of statins has less impact on the prognostic value of the TG/HDL-C ratio in diabetic patients with CAD. Several potential mechanisms may account for the association of the TG/HDL-C ratio with all-cause mortality and cardiovascular death in diabetic patients with CAD: (1) an elevated TG level and lower HDL-C plays an important role in endothelial dysfunction and atherosclerosis. Combined TG and HDL-C are significantly related to other atherogenic lipid phenotypes, characterized by higher levels of small dense LDL particles along with higher levels of remnant particle cholesterol and non-HDL-C, which contribute to the progression of atherosclerosis (14, 58, 59); (2) the TG/HDL-C ratio is significantly related to insulin resistance and glycemic control in diabetic patients (15, 16, 60, 61). Insulin resistance is related to the progression of atherosclerosis, vulnerability of coronary plaques, and MACEs in patients with CAD (62–64). Moreover, a hyperglycemic environment could induce the progression of macrovascular and microvascular disease in diabetic patients, including diabetic nephropathy, CAD and peripheral artery disease, which could cause excess risk of all-cause mortality and cardiovascular death (65, 66).

Additionally, the addition of the TG/HDL-C ratio in the risk prediction model for subsequent all-cause mortality and cardiovascular death was associated with a high predictive value. These results suggest that the use of TG/HDL-C ratio could refine risk stratification for all-cause mortality and cardiovascular death in diabetic patients with CAD who are treated with statins. Moreover, this study identified the optimal cutoff value of the TG/HDL-C ratio in this context, suggesting that the ratio should be maintained at <1.57 to reduce the risk of all-cause mortality and cardiovascular death. The results of this study provide new evidence to reduce all-cause mortality and cardiovascular death in diabetic patients with CAD treated with statins. Further large-scale prospective cohort studies should be performed to verify whether implementation of screening TG/HDL-C ratio will change the prognosis of diabetic patients with CAD.

However, several limitations of this study should be acknowledged. First, the current study was retrospective. The lack of information of waist circumference made it difficult to calculate the fatty liver index. Therefore, fatty liver index was not included in the analysis. Second, the follow-up information was collected by telephone or electronic medical record review. The follow-up information mainly included survival data. Baseline data after 4-year follow-up was not collected. Third, the information about glycemic control optimization and changes in medications was not collected during follow-up. The effect of changes in medications should be taken into consideration in the future, prospective study. Fourth, the complications and severity of T2DM and CAD differ, which could have affected the risk of all-cause mortality and cardiovascular death. Finally, as lipid levels vary among different ethnicities, it is not known whether these findings can be applicable to other ethnicities.

## Conclusion

An elevated TG/HDL-C ratio was associated with an increased risk of all-cause mortality and cardiovascular death in diabetic patients with CAD who were treated with statins. Moreover, the addition of the TG/HDL-C ratio into the traditional risk model increased the predictive value for subsequent all-cause mortality and cardiovascular death. Therefore, the TG/HDL-C ratio may be a useful marker for evaluating the prognosis in diabetic patients with CAD who are treated with statins.

## Data Availability Statement

The raw data supporting the conclusions of this article will be made available by the authors, without undue reservation.

## Ethics Statement

The studies involving human participants were reviewed and approved by the Ethical Committee of Tianjin Chest Hospital. The ethics committee waived the requirement of written informed consent for participation.

## Author Contributions

LW, HC, and JZ participated in the study design. LW, YH, AW, YZ, HY, LR, WQ, and WL participated in data collection. LW, HY, and LR performed the statistical analysis. LW drafted the article. All authors contributed to the article and approved the submitted version.

## Conflict of Interest

The authors declare that the research was conducted in the absence of any commercial or financial relationships that could be construed as a potential conflict of interest.
